# Polystyrene Nanoplastics Exposure Alters Gut Microbiota and Correlates with Egg Quality Parameters in Chickens

**DOI:** 10.3390/ani15213154

**Published:** 2025-10-30

**Authors:** Xuan Hu, Yinyin Liu, Wanqiang Chen, Yinping Ma, Yanfeng Fan, Qian Zhou, Mengmeng Lei, Hongsheng Song, Min Zhao, Xiaoxu Jia, Guodong Cai, Jianchun Bian, Yushi Gao

**Affiliations:** 1 Jiangsu Institute of Poultry Science, Yangzhou 225009, China; huxuan19970815@163.com (X.H.); 18901456756@163.com (Y.L.); chenwq8231@163.com (W.C.); 13665248923@163.com (Y.M.); fanyanfeng226@126.com (Y.F.); zhouqian_hx@163.com (Q.Z.); 18252719718@163.com (M.Z.); 13852787501@139.com (X.J.); 2 College of Veterinary Medicine, Yangzhou University, Yangzhou 225009, China; mz120241807@stu.yzu.edu.cn (M.L.); 13461126585@163.com (H.S.); 008400@yzu.edu.cn (G.C.); 3 Jiangsu Co-Innovation Center for Prevention and Control of Important Animal Infectious Diseases and Zoonoses, Yangzhou 225009, China

**Keywords:** NPs, chicken, 16s rRNA, cecal microbiota, diversity

## Abstract

NPs have become a concerning global environmental problem. Dietary exposure to NPs can cause microbial dysbiosis. However, the risks of NPs to animals, particularly poultry species such as chickens, remain poorly understood. In this study, chickens were continuously exposed to 100 nm NPs via dietary inclusion from 18 weeks of age for 120 days to evaluate the effects of NPs on intestinal health. We found that NPs accumulated in chicken intestinal tissues, leading to adverse alterations in the intestinal mucosal structure, such as villus atrophy and goblet cell depletion, and significantly altering intestinal length. The 16S rRNA sequencing revealed significant gut microbiota dysbiosis, characterized by a loss of diversity and shifts in key bacterial groups. Functional predictions of the microbiota revealed impairments in metabolic pathways, especially carbohydrate and amino acid metabolism. Furthermore, network analysis showed that microbial interactions were disrupted and key functional hubs were lost. Most importantly, NPs exposure led to a significant decline in egg quality parameters, including eggshell thickness and strength, yolk color, weight, shape index, and Haugh units. Correlation analyses connected specific taxa, such as *Methanobrevibacter*, *Rikenellaceae_RC9_gut_group*, and *Prevotellaceae_UCG-001*, to intestinal damage and declines in egg quality. These findings provide a scientific basis for assessing the health risks of NPs in animals and offer insights into the development of gut health interventions.

## Simple Summary

Plastic pollution is a growing global environmental threat, with nanoplastics (NPs) potentially posing risks to agricultural systems and food safety. Poultry may be exposed to NPs through contaminated feed; however, the impact of this exposure on the gut microbial community and its subsequent effects on egg quality are not fully understood. Our study examined the effects of dietary exposure to 100 nm NPs for 120 days by analyzing gut health, cecal microbiota, and egg quality. We found that NPs accumulated in the intestinal tract, causing structural damage such as villus shortening and a reduction in protective goblet cells. This was accompanied by a significant disruption of the gut microbiota, characterized by decreased diversity and shifts in key bacterial groups. Importantly, we observed reductions in eggshell thickness and strength, yolk color, egg weight, and Haugh units. Correlation analysis further linked these declines to specific changes in the gut microbial community. Our findings emphasize that gut health is a critical target of NPs toxicity. They highlight the essential role of a balanced gut microbiome in poultry productivity and provide valuable insights for developing strategies to reduce the impact of NPs on animal health and food quality.

## 1. Introduction

Plastic pollution has become widespread worldwide in recent years [1]. Plastics are accumulating in the environment at a rate of 31.9 million tons annually, with projections indicating that over 33 billion tons will have accumulated globally by 2050 [2]. Polystyrene (PS), a synthetic polymer compound that serves as a common raw material for plastics, is widely used in both consumer and industrial applications owing to its thermal stability, ease of manufacturing, and low cost [3]. Plastics can undergo progressive fragmentation into microplastics (MPs, less than 5 mm in diameter) or even smaller particles, known as nanoparticles (NPs, less than 100 nm in diameter). Furthermore, NPs are smaller than MPs and have a higher likelihood of crossing biological barriers that cause damage to the organism [4]. Therefore, in this study, we have focused on polystyrene nanoplastics (NPs) because of their ability to exert negative effects on aquatic animals and plants by accumulating in various animal organs and plant roots, thus indirectly posing a threat to animal health [5,6,7].

M/NPs are emerging environmental contaminants of great concern because of their ubiquitous distribution in air, soil, water, and food. Poultry are exposed to NPs through contaminated feed, water, and environmental ingestion [8]. The gastrointestinal tract, therefore, serves as the primary route of exposure, through which the gut microbial community is inevitably affected [7,9]. The intestinal microbiota plays a vital role in the physiological system of poultry, with significant effects encompassing host metabolism, immune function, health maintenance, and growth and development. These microorganisms play a crucial role in host growth and health through their functional contributions to digestion and absorption processes, with the intestine being one of the major organs where these processes occur [10,11]. The digestive tract is the main route of NPs exposure. Previous studies have found that NPs can affect the abundance and composition of the gut microbiota [12,13]. In aquatic models (e.g., zebrafish), NPs exposure disrupts gut microbiota composition and impairs carbohydrate and amino acid metabolism [14].

The pervasive presence of plastic pollution has raised alarms over its potential to infiltrate the food chain. The ability of NPs to traverse biological barriers and accumulate in animal tissues—as evidenced by their detection in human placenta [15]—poses a direct concern for the safety of animal-derived foods. This risk is particularly relevant to poultry production, as recent evidence confirming the presence of polystyrene microplastics in broiler feces underscores a tangible route of exposure [16]. As a vertebrate species occupying a high ecological niche, chickens and their products, such as eggs, are nutrient-rich and essential parts of daily life, playing a crucial role in food safety. Given that chickens and their eggs constitute a nutrient-rich staple of the global diet, contamination of these products could represent a significant pathway for human ingestion of plastic particles. While initial findings confirm exposure, the functional consequences of NPs ingestion on avian health and product quality remain poorly understood.

Therefore, this study was designed to bridge this knowledge gap by systematically investigating the effects of polystyrene NPs on chicken intestinal morphology and the cecal microbiota, assessing the impact of this exposure on a suite of egg quality parameters, and analyzing the correlative relationships between these microbial shifts and egg quality metrics.

## 2. Materials and Methods

### 2.1. Ethics Statement

All animal experiments were approved by the Animal Ethics Committee of the Jiangsu Institute of Poultry Science (Jiangsu, China; Approval No. 202411001). Euthanasia was carried out adhering to the American Veterinary Medical Association (AVMA) Guidelines for the Euthanasia of Animals (2020 edition), fulfilling institutional and national ethical standards for the humane use of animals in research.

### 2.2. NPs Characterization

Monodisperse NPs with a nominal diameter of 100 nm and a concentration of 25 mg/mL were procured from Tianjin Base-line ChromTech Research Centre (Tianjin, China). The stock suspension was diluted with double-distilled water (DDW) and subjected to sonication (40 kHz, 85% power, 10 min) in a water bath sonicator before each experiment to ensure homogeneity of dispersion. ATR-FTIR spectroscopic analysis of the NPs was conducted on a Nicolet iS50 spectrometer (Thermo Fisher Scientific, Waltham, MA, USA) to determine their chemical characteristics. For morphological analysis, the NPs were examined using scanning electron microscopy (SEM; Gemini SEM 300, Oberkochen, Germany), which allowed direct visualization of NPs morphology, size, and dispersion state at the nanoscale, confirming both primary particle size and the absence of large aggregates.

### 2.3. Experimental Animals

A total of 60 green-shelled laying hens (120 days old) underwent a 7-day acclimation period under standardized environmental conditions. All birds were housed in individual cages under a standardized lighting schedule of 16h of light and 8h of dark, with light intensity maintained at approximately 50 lux. The temperature was maintained at 24 ± 1 °C and the relative humidity at 55 ± 5%. Birds had ad libitum access to feed and water throughout the experimental period. The birds were randomly allocated into two experimental groups (*n* = 30 per group). Based on evidence that the aquatic environment serves as a major route of microplastic exposure in avian species, the exposure concentration used in this study was selected to reflect environmentally relevant levels as reported in previous research [17]. One group received a basal diet (for detailed composition, see Table S1) supplemented with 100 nm NPs at a concentration of 10 mg/kg feed, while the control group received the same basal diet without NPs. The experimental period spanned 120 days, during which feed and water were provided ad libitum. After a 12 h fasting period, all birds were humanely euthanized by CO_2_ asphyxiation. During dissection, cecal and fecal contents were collected. Tissue samples designated for histopathological examination were immediately fixed in 4% paraformaldehyde, and those intended for microbial analysis were flash-frozen in liquid nitrogen and stored at −80 °C until DNA extraction.

### 2.4. Ex Vivo Fluorescence Imaging

Fluorescent imaging was performed on chickens using an IVIS Lumina III in vivo imaging system (PerkinElmer, Shelton, CT, USA). The acquisition parameters were set to an excitation wavelength of 580 nm and an emission wavelength of 620 nm. Following image acquisition, fluorescence intensity was quantified using Living Image Software 4.5.2 (PerkinElmer, USA). Regions of interest (ROIs) were manually delineated over the abdominal area, which was selected as the primary region for NPs accumulation based on gastrointestinal tract anatomy. The total radiant efficiency ([*p*/*s*]/[μW/cm^2^]) was calculated for each ROI. To ensure accuracy, background fluorescence signals obtained from the same-sized ROIs in control animals not exposed to NPs were subtracted from all measurements.

### 2.5. Hematoxylin and Eosin Staining

All chicken intestines were surgically removed, trimmed, and immersed in a fixative solution containing 4% paraformaldehyde for 24 h, by established protocols. After discarding the fixative, the tissue was cleaned, allowed to dry naturally using a gradient of ethanol concentration, turned transparent using a xylene solution, and then impregnated with wax. Sections of the tissue were attached to slides using a microtome. HE was used to stain the cell nucleus and cytoplasm of tissue wax specimens directly sectioned at a thickness of 5 µm. Every specimen was examined and documented utilizing a Leica light microscope DMI3000B (Leica, Wetzlar, Germany) outfitted with a digital camera.

### 2.6. Periodic Acid Schiff Staining

Tissue sections were treated with periodic acid solution (5 g/L in distilled water) for 5 min, followed by rinsing with tepid distilled water for 5 min. Subsequently, slides were stained with Schiff reagent for 5 min and counterstained with hematoxylin for 2 s. After staining, sections were dehydrated through an ethanol series, cleared in xylene, and mounted. For microscopic analysis of goblet cells in chicken intestinal tissues, the stained sections were examined using a Leica DMI3000B system (Wetzlar, Germany).

### 2.7. Measurement of Egg Quality Parameters

Egg quality on study day 120 was evaluated. Six eggs from each group were collected within 24 h for analysis. Parameters were measured using the following methods and instruments. Eggshell thickness was measured using the ETG-1061A instrument (Robotmation, Tokyo, Japan) with measurements taken at three positions (blunt end, equatorial region, and sharp end) and the average value calculated. Egg shape index was determined using a Digimatic caliper (0–200 mm; Guilin Guanglu, Guilin, China) by measuring the maximum vertical diameter and maximum horizontal diameter, with the index calculated as (maximum vertical diameter/maximum horizontal diameter) × 100. Eggshell strength was assessed using the EFG-0502 tester (Robotmation, Japan) by applying force to the equatorial region of the egg until fracture occurred. Egg white height, yolk color, and Haugh units were collectively measured using the EMT-7300 multi-functional tester (Robotmation, Japan). The Haugh unit was calculated using the standard formula HU = 100 × log (H − 1.7 W^0.37 + 7.6), where H is the albumen height (mm) and W is the egg weight (g).

### 2.8. 16S rRNA Sequencing

Fecal samples were collected from the chickens in each group. According to the manufacturer’s protocols, total microbial genomic DNA was extracted from the fecal samples using the TIANamp Soil DNA Kit (TianGen, Beijing, China). The bacterial primers 341F (5′-CCTAYGGGRBGCASCAG-3′) and 806R (5′-GGACTACNNGGGTATCTAAT-3′) were used to amplify the V3–V4 hypervariable regions of the 16S rRNA gene. All PCR reactions were performed using 15 μL of Phusion High-Fidelity PCR Master Mix, 0.2 μM of each forward and reverse primer, and approximately 10 ng of template DNA. Thermal cycling consisted of an initial denaturation at 98 °C for 1 min, followed by 30 cycles of denaturation at 98 °C for 10 s, annealing at 50 °C for 30 s, elongation at 72 °C for 30 s, and a final extension at 72 °C for 5 min. The PCR products were purified using a magnetic bead-based method.

The amplicon libraries were sequenced on an Illumina NovaSeq 6000 platform (Illumina, San Diego, CA, USA) using a 2 × 250 bp paired-end configuration. Raw sequencing data were processed using QIIME 2 (version 2023.9). Sequence denoising, including quality filtering, dereplication, chimera removal, and paired-end read merging, was performed with the DADA2 plugin, which generated amplicon sequence variants (ASVs) as the final features. For subsequent diversity analyses, all samples were rarefied to a depth of 30,000 sequences per sample to ensure even sampling depth. Both α-diversity (Shannon, Simpson, Chao1, and observed features) and β-diversity (weighted and unweighted UniFrac, Bray–Curtis) metrics were calculated based on this rarefied ASV table.

Alpha Diversity

To analyze the diversity, richness, and uniformity of the communities in the sample, alpha diversity was calculated from 7 indices in QIIME2(version 202202), including Observed_otus, Chao1, Shannon, Simpson, Dominance, and Pielou_e. Three indices were selected to identify community richness: Observed_otus—the number of observed species (https://www.osgeo.cn/scikit-bio/generated/skbio.diversity.alpha.observed_otus.html (accessed on 15 June 2025)); Chao—the Chao1 estimator (https://www.osgeo.cn/scikit-bio/generated/skbio.diversity.alpha.chao1.html#skbio.diversity.alpha.chao1 (accessed on 15 June 2025)); and Dominance—the dominance index (https://www.osgeo.cn/scikit-bio/generated/skbio.diversity.alpha.dominance.html#skbio.diversity.alpha.dominance (accessed on 15 June 2025)). Two indices were used to identify community diversity: Shannon—the Shannon index (https://www.osgeo.cn/scikit-bio/generated/skbio.diversity.alpha.shannon.html#skbio.diversity.alpha.shannon (accessed on 15 June 2025)) and Simpson—the Simpson index (https://www.osgeo.cn/scikit-bio/generated/skbio.diversity.alpha.simpson.html#skbio.diversity.alpha.simpson (accessed on 15 June 2025)). One index was used to calculate sequencing depth: coverage—the Good’s coverage (https://www.osgeo.cn/scikit-bio/generated/skbio.diversity.alpha.goods_coverage.html#skbio.diversity.alpha.goods_coverage (accessed on 15 June 2025)); One index was used to estimate species evenness: Pielou_e—Pielou’s evenness index (https://www.osgeo.cn/scikit-bio/generated/skbio.diversity.alpha.pielou_e.html#skbio.diversity.alpha.pielou_e (accessed on 15 June 2025)). To evaluate the richness of the microbial community and sample size, a species accumulation boxplot can be used to visualize data, which is performed with the vegan package in R software.

Beta Diversity

Beta diversity was evaluated through cluster analysis. Before clustering, principal component analysis (PCA) was employed for dimensionality reduction of the original variables using the ade4 and ggplot2 packages in R software (version 4.0.3). Principal coordinates analysis (PCoA) was subsequently performed to extract principal coordinates and visualize complex, multidimensional data. A distance matrix based on weighted or unweighted UniFrac metrics was constructed and projected onto a new set of orthogonal axes, where the first principal coordinate represents the maximum variance, followed by successively lower variances in subsequent coordinates. All PCoA visualizations were generated using the ade4 and ggplot2 packages in R software. Non-metric multidimensional scaling (NMDS) was also implemented for data dimension reduction. Like the PCoA, NMDS also uses the distance matrix, but it emphasizes the numerical rank instead. The distance between sample points on the diagram can only reflect the rank information rather than the numerical differences. NMDS analysis was implemented using the R software with the ade4 and ggplot2 packages.

### 2.9. Quantification and Statistical Analysis

Statistical analyses were performed using SPSS (version 22.0; IBM, Armonk, NY, USA) and R software (version 4.3.1). The normality of all data was examined using the Kolmogorov–Smirnov test. For comparisons between the two groups, data conforming to a normal distribution were analyzed using Student’s *t*-test; results are expressed as the means ± standard deviation. *p* < 0.05 indicates a significant difference. Spearman’s correlation analysis was used to examine relationships between specific microbial genera and egg quality parameters. The Mann–Whitney U test was used for the predictive analysis of gut microbiota functions.

## 3. Results

### 3.1. Morphological and Chemical Characterization of NPs

**Figure 1:**
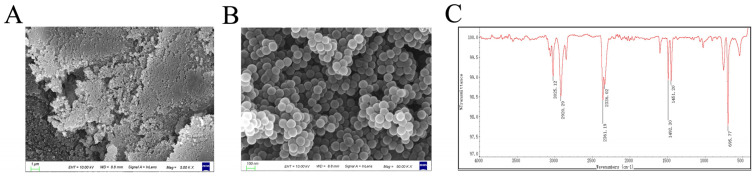
Morphological and chemical characterization of NPs. (**A**) Representative scanning electron microscopy (SEM) image of NPs at a 1 μm scale. (**B**) High-magnification SEM image showing detailed NP morphology at a 0.1 μm scale. (**C**) Fourier-transform infrared (FTIR) spectrum of NPs showing characteristic absorption bands.

The morphological characteristics of the NPs were characterized by scanning electron microscopy (SEM). SEM images (Figure 1A,B) revealed monodisperse nanoparticles with smooth surfaces and uniform spherical morphology. The nanoparticle suspension was homogeneously dispersed with well-defined boundaries. The particle size was determined to be in the range of 80–100 nm. Fourier-transform infrared (FT-IR) spectroscopy (Figure 1C) confirmed the chemical identity of the NPs, with absorption peaks corresponding to characteristic vibrational modes of the polystyrene benzene ring.

### 3.2. Exposure to Organ Coefficients and Morphology of Cecal Microbiota

**Figure 2:**
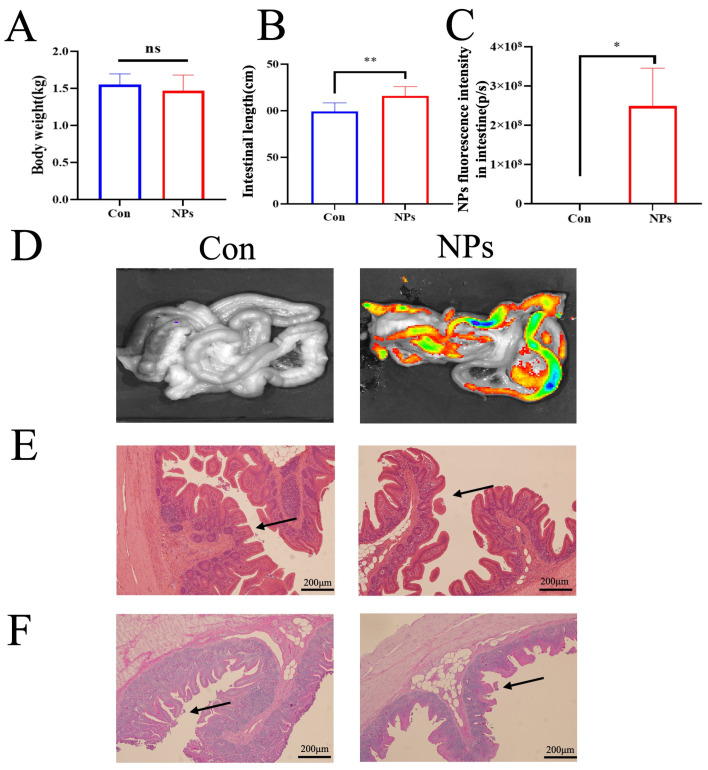
Effects of NPs on growth parameters and intestinal morphology between the Con and NPs groups. (**A**) Body weight measurements. (**B**) Intestinal length measurements. (**C**) Quantification of NPs fluorescence intensity in intestinal tissues. (**D**) Representative fluorescence images showing the NPs distribution in chicken intestinal tissues. (**E**) Hematoxylin and eosin (HE) staining of intestinal tissue sections across experimental groups (scale bars = 200 µm, black arrowheads indicate intestinal villi). (**F**) Periodic acid-Schiff (PAS) staining of intestinal tissue sections demonstrating goblet cell distribution (scale bars = 200 µm, black arrowheads indicate intestinal villi). Data are presented as mean ±standard deviation (SD) (*n* = 6 per group). Statistical significance compared to the Con group. ns = not significant; * *p* < 0.05, ** *p* < 0.01.

To assess the impacts of NPs on intestinal health, body weight, intestinal length, fluorescence imaging, and histopathological features were evaluated. No significant differences in body weight were observed between the Con and NPs groups (*p* = 0.3343; Figure 2A). However, significant alterations in intestinal length were detected in the NPs group compared to the Con group (*p* = 0.0009; Figure 2B). Fluorescence imaging showed markedly greater accumulation of NPs in the intestinal tissues of chickens administered NPs compared to the Con group (*p* = 0.0110; Figure 2C,D). Histopathological examination revealed structural abnormalities, including sparse microvilli, villus shortening, disruption of epithelial tight junctions (Figure 2E), and a reduction in goblet cell density compared to the Con group (Figure 2F). These results demonstrate that NPs exposure can adversely affect intestinal integrity and healthy development in chickens.

### 3.3. Effects of NPs Exposure on Egg Quality

**Figure 3:**
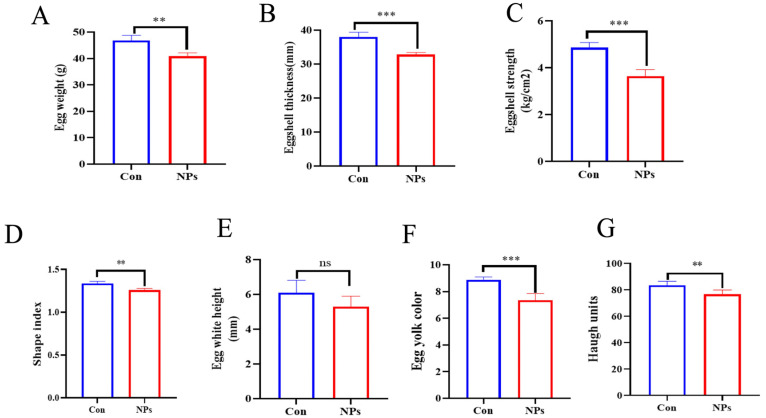
Effects of NPs exposure on egg quality parameters between groups Con and NPs. (**A**) Egg weight, (**B**) eggshell thickness, (**C**) eggshell strength, (**D**) egg shape index, (**E**) egg white height, (**F**) egg yolk color, (**G**) Haugh units. Data are presented as mean ± standard deviation (SD) (*n* = 6 per group). Statistical significance was determined using Student’s *t*-test and is indicated as follows: ns = not significant; ** *p* < 0.01, *** *p* < 0.001.

We assessed the effect of NPs exposure on egg quality by comparing the NPs-treated group with the Con group. NPs exposure significantly impaired several key egg quality parameters: egg weight and shape index were markedly decreased (Figure 3A,D; *p* = 0.0001). Similarly, eggshell thickness, eggshell strength, and egg yolk color were also significantly reduced (Figure 3B,C,F; *p* < 0.0001). Furthermore, Haugh units were significantly lower in the NPs group compared to the control (Figure 3G; *p* = 0.0037). In contrast, no significant difference was observed in egg white height between the groups (Figure 3E; *p* = 0.0632). These results indicate that exposure to NPs impaired key production traits related to eggshell formation, yolk composition, and albumen quality.

### 3.4. Effect of NPs Exposure on Cecal Microbial Community Composition and Diversity

**Figure 4:**
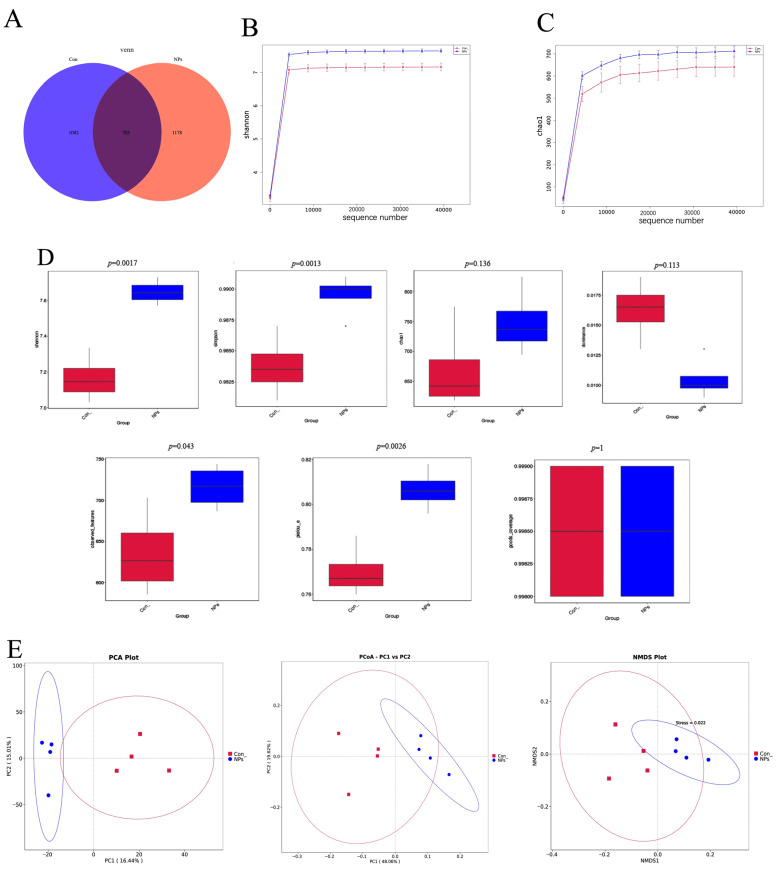
Differences in cecal microbiota composition and diversity between Con and NPs Groups. (**A**) Characteristics of sequence statistics for the Con and NPs groups. (**B**,**C**) Overall richness and diversity statistics for the Con and NPs groups. (**D**) Shannon, Simpson, Chao1, dominance, observed_features, Pielou_e, and goods_coverages box plot illustrating Con and NPs group differences in α-diversity indices. (**E**) PCA, PCoA, and NMDS plot illustrating Con and NPs group differences in β-diversity analysis. All ordination plots were generated using the weighted UniFrac distance metric.

To investigate the effects of NPs exposure on the cecal microbiota, we performed high-throughput 16S rRNA gene sequencing of cecal contents from Con and NPs-treated groups. Sequencing generated 1,228,156 raw reads with an average of 102,346 reads per sample, and quality control showed Q20 > 98% and Q30 > 93% across all datasets (Table S2). A total of 2295 feature sequences were identified, with 1082 and 1178 features unique to the Con and NPs groups, respectively, and 705 features shared between groups (Figure 4A). Rarefaction curves plateaued, indicating sufficient sequencing depth to capture microbial richness and diversity in all samples. Comparative analysis revealed significant differences in cecal microbial communities between Con and NPs-treated groups (Figure 4B,C). These results demonstrate reliable sequencing data, enabling subsequent analyses.

This compositional divergence was reflected in significant alterations of microbial diversity. The α-diversity, measured by seven indices, showed significant differences between groups for the Shannon, Simpson, observed features, and Pielou’s evenness indices (*p* < 0.05), while no significant differences were observed in the Chao1, dominance, and Good’s coverage indices (Figure 4D). Furthermore, β-diversity analysis (PCoA, PCA, NMDS) based on weighted UniFrac distances confirmed a clear separation between the two groups (Figure 4E). Collectively, these results demonstrate that NPs exposure significantly alters both the composition and structure of the cecal microbiota in chickens.

### 3.5. Effect of NPs Exposure on Cecal Microbial Community Composition

**Figure 5:**
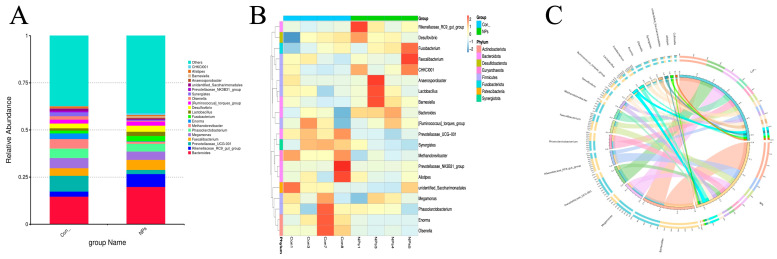
Compositional profiling analysis of cecal microbial differences between the Con and NPs groups. (**A**) Relative abundance of the top 20 bacterial genera. (**B**) Heatmap of the top 20 abundant phyla in cecal microbiota. (**C**) Circos plot of sample-species associations (top 20 taxa; line width proportional to abundance).

We profiled the bacterial community at the genus level in both Con and NPs groups to further investigate the specific compositional characteristics of the chicken intestinal microbiota. At the genus level, the top 20 genus were identified, with *Bacteroides* and *Rikenellaceae_RC9_gut_group* being the most abundant families (Figure 5A, Table S3). In contrast, *Prevotellaceae_UCG-001* exhibited significantly reduced abundance in the NPs groups versus the Con. The beneficial butyrate-producing genus *Faecalibacterium* maintained comparable colonization levels between groups. Hierarchical clustering analysis (Figure 5B, Table S4) confirmed distinct segregation patterns among these differentially abundant taxa. Circos plot visualization (Figure 5C and Table S5) demonstrated robust intergroup associations for *Bacteroides* and *Rikenellaceae_RC9_gut_group*, as evidenced by connection bandwidths exceeding 3 mm. These findings suggest that these taxa may serve as key discriminative biomarkers and potential mediators of the observed phenotypic variations between experimental groups.

### 3.6. Effect of NPs Exposure on Species-Level Taxonomic Correlations and Statistical Tests

**Figure 6:**
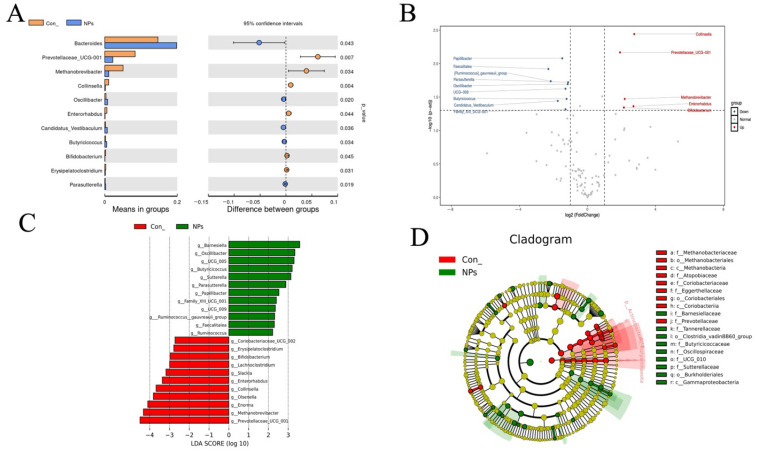
Statistical and phylogenetic analysis of cecal microbial differences between Con and NPs groups. (**A**) Analysis of species differences between groups using a *T*-test (*p* < 0.05). (**B**) Volcano plot displaying differentially abundant species (|log~2~FC| > 1, FDR-adjusted *p* < 0.05). Points above the x-axis represent species with higher abundance in the first comparison group (up), while points below indicate higher abundance in the second group (down). (**C**) LEfSe, the criterion of differential taxa was LDA scores > 2. Evolutionary Branch Diagram. (**D**) Phylogenetic cladogram demonstrating the evolutionary distribution of discriminant taxa (LDA score > 2). Circle diameter corresponds to relative abundance.

To investigate the effects of NPs exposure on the cecal microbial community, we employed a *t*-test algorithm to identify the microbial species that showed significant differences between the Con and NPs groups, and these species could serve as biomarkers. Differential abundance analysis identified 17 significant microbial biomarkers distinguishing the Con and NPs groups (*p* < 0.05, Figure 6A). These findings were validated through volcano plot analysis, which confirmed substantial alterations in microbial abundance profiles (Figure 6B). The following taxa were significantly upregulated: *Prevotellaceae_UCG-001*, *Methanobrevibacter*, *Enterorhabdus*, *Collinsella* and *Bifidobacterium*. Downregulated taxa included *Bacteroides*, *Oscillibacter*, *Candidatus_Vestibaculum*, *Butyricicoccus*, *Erysipelatoclostridium*, *Parasutterella*, *Family_XIII_UCG-001*, *Faecalitalea*, *[Ruminococcus]_gauvreauii_group*, *Papillibacter*, and *Ruminococcus*. Meanwhile, we also used LEfSe analysis to comprehensively identify differential taxa associated with NPs exposure (Figure 6C,D, LDA score > 2, *p* < 0.05). The LDA bar plot highlighted that the Con was dominated by the following groups: Coriobacteriaceae_UCG_002, Erysipelatoclostridium, Bifidobacterium, Lachnoclostridium, Slackiaceae, *Enterorhabdus*, *Collinisella*, *Olserjella*, *Enorma*, *Methanobrevibacter*, and *Prevotellaceae_UCG_001*. The NPs group was enriched in *Barnesiella*, *Oscillibacter*, *Butyricicoccus*, *Sutterella*, *Parasutterella*, *Papillibacter*, *Family_XIII_UCG_001*, *[Ruminococcus]_gauvreauii_group*, *Faecalitalea*, and *Ruminococcus*. These findings at the species level consistently identified specific microbial taxa altered by NPs exposure, providing reliable biomarkers for elucidating the mechanisms underlying gut health impairment.

### 3.7. Effect of NPs Exposure on Functional Enrichment Analysis of Cecal Microbiota

**Figure 7:**
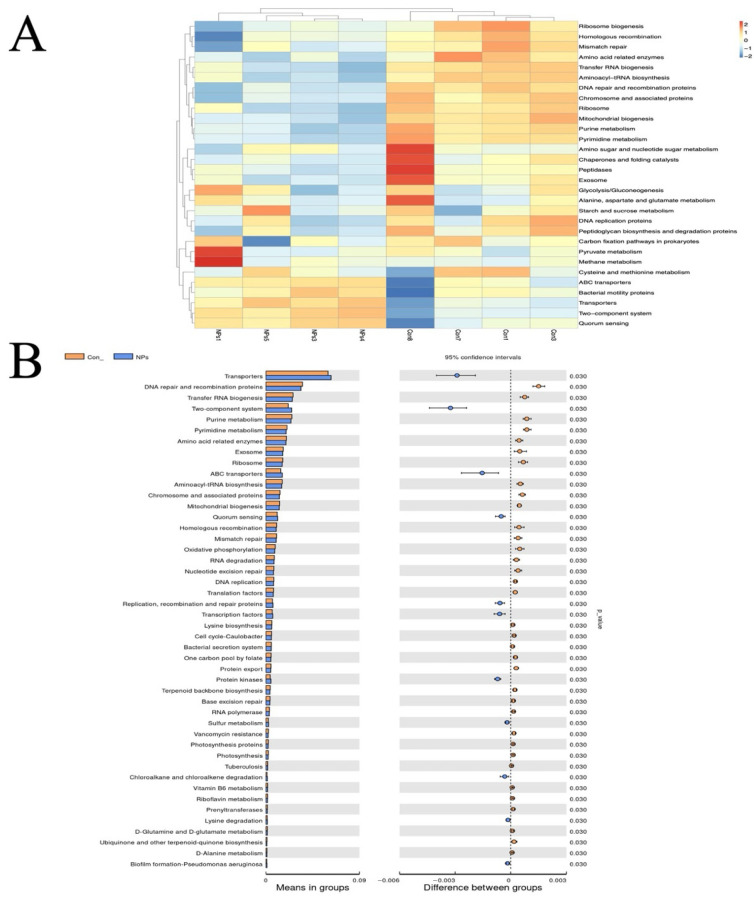
Tax4Fun-based functional annotation analysis of cecal microbial differences between the Con and NPs groups. (**A**) Heatmap of KEGG pathway abundances. Columns represent samples; rows show KEGG orthologs (KO). The left dendrogram indicates functional clustering. (**B**) Differential functional analysis. Left, group-wise mean abundance of significantly different KEGG pathways (*t*-test, *p* < 0.05). Right, 95% confidence intervals (circles) and *p*-values (color-coded) for mean differences.

To predict functional impacts of cecal microbiota on the Con and NPs groups, we used Tax4Fun-based functional annotation on the KEGG database. This identified 5927 KEGG orthologs (KOs) corresponding to 377 pathways. Pathway enrichment analysis revealed that microbiota-associated functions were predominantly metabolic (primary level), followed by genetic information processing, environmental information processing, cellular processes, human diseases, and organismal systems. Dominant secondary classifications included carbohydrate metabolism, replication and repair, translation, membrane transport, and amino acid metabolism. The top 30 most abundant level 3 pathways were selected for heatmap visualization, clustered by functional category (Figure 7A). Comparative analysis identified significantly enriched pathways (*p* < 0.05) in transporters, DNA repair/recombination proteins, tRNA biogenesis, two-component systems, pyrimidine metabolism, amino acid-related enzymes, exosome, ribosome, and ABC transporters (Figure 7B).

### 3.8. Effect of NPs Exposure on Correlation Analysis of Cecal Microbiota with Weight and Egg Quality-Related Traits

**Figure 8:**
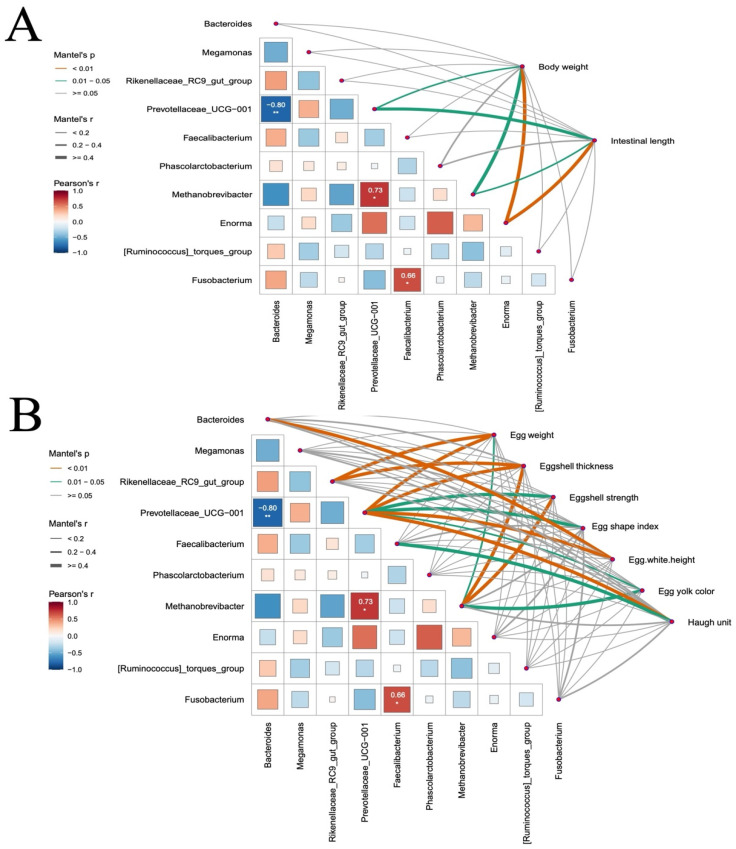
Correlation analysis of cecal microbiota with weight and egg quality between the Con and NPs groups. (**A**) Correlation analysis at the genus level between cecal microbiota and both body weight and intestinal length in chickens. (**B**) Correlation analysis at the genus level between cecal microbiota and multiple egg quality parameters, including egg weight, eggshell thickness, eggshell strength, egg shape index, albumen height, yolk color, and Haugh unit. * *p* < 0.05 indicates differences; ** *p* < 0.01 indicates significant differences; *** *p* < 0.001 indicates extremely significant differences.

To examine correlations between cecal microbiota, body weight, and egg quality in chickens, we performed Spearman correlation analysis using the linkET package, assessing microbial taxa from the cecal microbiota of the Con and NPs groups alongside productivity indicators that differed between groups including body weight, intestinal length, egg weight, eggshell thickness, eggshell strength, egg shape index, egg white height, yolk color, and Haugh unit. At the genus level, *Prevotellaceae_UCG-001* and *Methanobrevibacter* (*R* = 0.73, *p* < 0.05), and *Faecalibacterium* and *Fusobacterium* exhibited a significant positive correlation (*R* = 0.66, *p* < 0.05); *Prevotellaceae_UCG-001* and *Bacteroides* exhibited a significant negative correlation (*R* = −0.8, *p* < 0.01). Mantel test correlation analysis revealed significant positive correlation between body weight and *Enorma* (*R* = 0.52, *p* = 0.007), intestinal length and *Enorma* (*R* = 0.59, *p* = 0.009), body weight and *Methanobrevibacter* (*R* = 0.43, *p* = 0.014), intestinal length and *Methanobrevibacter* (*R* = 0.34, *p* = 0.043), body weight and *Prevotellaceae_UCG-001* (*R* = 0.33, *p* = 0.05), intestinal length and *Prevotellaceae_UCG-001* (*R* = 0.42, *p* = 0.025) (Figure 8A). Significant positive correlations with egg quality parameters included all egg quality parameters and *Prevotellaceae_UCG-001*, egg weight and *Rikenellaceae_RC9_gut_group* (*R* = 0.46, *p* = 0.001), eggshell thickness and *Rikenellaceae_RC9_gut_group* (*R* = 0.51, *p* = 0.006), egg weight and *Methanobrevibacter* (*R* = 0.31, *p* = 0.049), eggshell thickness and *Methanobrevibacter* (*R* = 0.56, *p* = 0.003), eggshell strength and *Methanobrevibacter* (*R* = 0.49, *p* = 0.002), egg yolk color and *Methanobrevibacter* (*R* = 0.55, *p* = 0.012), egg white height and *Bacteroides* (*R* = 0.61, *p* = 0.005), and Haugh unit and *Faecalibacterium* (*R* = 0.52, *p* = 0.023) (Figure 8B).

## 4. Discussion

Plastic pollution has become an environmental problem that cannot be ignored. In aquatic environments, plastic debris causes entanglement and ingestion hazards to marine life, while NPs can accumulate in sediment and water columns, disrupting nutrient cycles and food webs. Current research has primarily focused on its impacts on aquatic organisms and plants [14], with limited investigation into its accumulation and toxicological effects in terrestrial species, particularly in the context of the food chain. Chickens, like humans, are exposed to NPs through contaminated feed, mimicking real-world exposure routes such as leaching from food packaging (e.g., plastic bags, tea bags, etc.) or bioaccumulation in agricultural products [18,19]. This study offers new insights into the effects of NPs on poultry intestinal health and gut microbiota. We provide compelling evidence that NPs cause gut microbiota dysbiosis, impair metabolic function, and reduce egg quality. These findings expand research on plastic particle toxicity from aquatic systems to terrestrial food-producing animals and have important implications for animal health and food safety.

SEM and FTIR analyses confirmed the successful synthesis of monodisperse NPs with uniform spherical shape (100 nm) and characteristic polystyrene chemical bonds. The uniform dispersion and clear edges of the NPs suggest minimal aggregation, which is vital for assessing their biological interactions. We therefore sought to determine the in vivo distribution and toxicological consequences of these NPs within the intestinal tract. Increasing evidence has suggested that MPs can accumulate in the gut and cause intestinal damage. For example, MPs caused oxidative damage and inflammation in the gut, and destroyed all kinds of intestinal barriers, including physical barriers, chemical barriers, microbiological barriers, and immune barriers [20]. Our findings demonstrate that 100 nm NPs preferentially accumulate in intestinal tissues, disrupt villus architecture, and decrease the number of mucin-producing goblet cells. However, previous studies have mainly focused on the hazardous effect of NPs on the healthy intestine, while the responses of the intestine to NP upon certain kinds of stress may differ from those of healthy intestines [18,21]. This physical damage to the intestinal tract is expected to significantly alter the gut microbiome, thereby impacting the resident microbial community.

The gut microbiota is a complex and dynamic microecosystem that is essential for food metabolism, nutrient absorption, and maintaining intestinal barrier integrity. The natural stability of the gut microbiota results from interactions and plasticity within the microbial community [22]. However, certain factors, especially MPs, can disrupt the intestinal environment and impair the survival of the microbiota [23]. The total OTU count corresponds to microbial species richness, while the Shannon and Simpson indices characterize microbial diversity within samples. The Chao1 index reflects community richness by estimating total species abundance. Our 16S rRNA sequencing results demonstrate that NPs exposure significantly alters the cecal microbiota composition in chickens, consistent with previous reports of microbial community disruption following MPs exposure [24,25]. The observed reduction in shared feature sequences (30.7%) between the Con and NPs groups provides direct evidence for NPs-induced microbial restructuring, supporting findings by Deng et al. regarding MP-associated dysbiosis [24]. These microbial alterations may have functional consequences, as maintenance of gut microbial homeostasis is known to be critical for proper intestinal function, including nutrient absorption and barrier maintenance [26,27]. The observed microbial shifts could therefore contribute to increased intestinal permeability and metabolic disturbances, though further research is needed to establish direct causal relationships in avian models. Meanwhile, this may also be one of the reasons for the decreasing growth performance of chickens during exposure to NPs. In addition, we observed significant changes in the major components of the gut microbiota between the groups. These results demonstrate that gut microbial homeostasis is strongly influenced by NPs.

Having established these broad changes in microbial community structure, we then used network analysis to identify the specific keystone taxa and interactions most affected by NPs exposure. Network analysis showing Bacteroides and *Faecalibacterium* as high-connectivity hubs is particularly noteworthy, as these taxa are known to play essential roles in maintaining microbial community stability and host metabolic health [28]. The preservation of *Faecalibacterium* abundance despite considerable community restructuring may indicate a compensatory mechanism to sustain butyrate production, which is vital for colonocyte health and anti-inflammatory effects. However, the significant reduction in other beneficial taxa such as *Butyricicoccus* and *Ruminococcus* suggests a potential impairment of microbial functions related to fiber fermentation and short-chain fatty acid production. The identification of 17 significant microbial biomarkers through LEfSe analysis provides strong evidence for the selective pressure exerted by NPs on gut microbiota composition. These findings support the idea that MPs exposure not only directly impacts microbial abundance but also disrupts the complex network of microbial interactions responsible for maintaining gut homeostasis.

To determine the functional implications of this disrupted microbial network, we performed functional prediction based on 16S rRNA gene sequencing data using the Tax4Fun tool [29]. The observed alterations in predicted metabolic pathways may be functionally linked to the specific taxonomic shifts reported previously. Notably, the predicted impairments in carbohydrate and amino acid metabolism pathways could be partially attributed to the reduction in *Prevotellaceae_UCG-001*, a taxonomic group recognized for its involvement in polysaccharide degradation. The concurrent predicted downregulation of ABC transporters is also consistent with a potential decrease in nutrient absorption capacity [30]. Meanwhile, the predicted upregulation of DNA repair pathways and two-component systems suggests an activated microbial stress response, which appears to correlate with the enrichment of *Bacteroides*, a genus often observed to exhibit resilience under stress conditions [31,32]. Furthermore, the predicted differential abundance of genes related to ribosomal proteins and tRNA biogenesis indicates a potential restructuring of microbial protein synthesis, which might contribute to the observed community-wide taxonomic changes. These metabolic disruptions likely contribute to the compromised intestinal barrier function and reduced nutrient utilization efficiency observed in NPs-exposed poultry [33], highlighting the need for further investigation into targeted interventions to maintain microbial homeostasis in agricultural systems.

To directly link these predicted functional impairments to the observed decline in egg quality, we performed correlation analysis between microbial taxa and egg quality parameters. Our results identify the decline of *Prevotellaceae_UCG-001*, a bacterial genus whose abundance correlated positively with all egg quality metrics, as a key mediator in the pathway through which NPs exposure compromises egg quality. This genus represents primary degraders of dietary fiber, which are responsible for generating short-chain fatty acids (SCFAs) through fermentation. In laying hens, SCFAs serve as essential energy substrates and provide critical carbon skeletons for vitellogenesis (yolk formation) and eggshell biomineralization [34]. Furthermore, the increased abundance of *Bacteroides* and its positive correlation with albumen height may indicate a metabolic shift towards enhanced utilization of intestinal mucins or dietary proteins [35]. Additionally, the strong positive correlations between *Methanobrevibacter* and eggshell quality metrics suggest that NPs may disrupt microbial cross-feeding interactions, particularly hydrogen transfer, thereby impairing overall metabolic efficiency [36]. Collectively, these findings indicate that NPs induce a comprehensive decline in egg quality by disrupting a core functional network of gut microbiota that is essential for host nutrient utilization.

Collectively, our findings underscore the pivotal role of gut microbiota in poultry health and production. Accumulating evidence implies the intestinal microbiota of laying hens is a potential mediator to improve prevalent issues in terms of egg quality decline in the late phase of laying production [33]. Our results demonstrate that NPs exposure initiates a pathological cascade from intestinal damage to systemic productivity loss in chickens. Gut barrier disruption and microbial dysbiosis directly drive multifaceted egg quality impairment: Eggshell integrity deterioration aligns with suppressed *Methanobrevibacter* abundance, as evidenced by positive correlations with strength metrics. Yolk/albumen quality decline corresponds to *Faecalibacterium* depletion and *Bacteroides* enrichment. These findings establish gut microbiota as a pivotal regulator of NPs-induced productivity loss, providing a foundation for microbial homeostasis-targeted interventions.

In conclusion, this study provides clear evidence that NPs induce intestinal damage and microbial dysbiosis in broilers, characterized by reduced *Bacteroides* abundance and compromised egg quality. *Bifidobacterium* depletion correlates with impaired nutrient absorption efficiency, while elevated *Escherichia-Shigella* abundance associates with proinflammatory responses and growth reduction. These alterations establish NPs-induced gut microbiota disruption as a primary driver of egg quality decline and systemic productivity loss in poultry systems.

## 5. Conclusions

In summary, this study demonstrates that NPs exposure impairs poultry intestinal integrity via concurrent physical damage and microbial dysregulation. Results confirm intestinal NPs accumulation occur alongside significant reductions in microbial diversity and compositional shifts. These findings refine NPs risk assessment in agriculture and inform targeted strategies for poultry intestinal health management to safeguard productivity.

## Data Availability

All data generated or analyzed during this study are included in this published paper.

## Supplementary Materials

The following supporting information can be downloaded at https://www.mdpi.com/article/10.3390/ani15213154/s1: Table S1. Composition and nutrient levels of the basal diet. Table S2. 16s rRNA Data quality control. Table S3. 16s rRNA averaged relative abundance of the top 20 main intestinal microbial colonies at the genus level. Table S4. Relative abundance of 16s rRNA clustering heatmap. Table S5. 16s rRNA relative circos of the top 20 main intestinal microbial colonies at the genus level.
