# Supplementary material for: Polystyrene Nanoplastics Exposure Alters Gut Microbiota and Correlates with Egg Quality Parameters in Chickens
Source: Animals (Basel). 2025 Oct 30;15(21):3154. doi: 10.3390/ani15213154 (PMC12606741; doi:10.3390/ani15213154)
Supplement: Supplementary file 1 [file animals-15-03154-s001.zip › animals-3895722-supplementary.pdf]

animals

MDPI

Supplementary materials:

Table S1. Composition and nutrient levels of the basal diet.

Item	Content	Nutrient Levels	Content
Ingredients		Nutrients	
Corn	62.8%	Metabolizable Energy, MJ/kg	11.3 MJ/kg
Soybean Meal	26.2%	Crude Protein	16.50%
Limestone	8.87%	Crude Fiber	2.74%
Wheat Bran	0.5%	Calcium	3.68%
Soybean Oil	0.5%	Available Phosphorus	0.34%
Dicalcium Phosphate	1.2%	Methionine	0.42%
Methionine	0.13%	Lysine	0.85%
Premix	0.5%		
Total	100		

Table S2. 16s rRNA Data quality control.

Group	Sample	RawPE	Combined	Qualified	Nochime	Base(nt)	Avglen(nt)	GC	Q20	Q30
Con	Con 1	104319	103500	100361	70660	29469900	417.07	55.00%	98.14%	93.98%
	Con 2	103761	102949	99892	72761	30244739	415.67	54.09%	98.32%	94.29%
	Con 3	104975	104289	102062	76497	31886830	416.84	55.75%	98.49%	94.84%
	Con 4	103044	102268	100068	62612	26122144	417.21	56.33%	98.37%	94.59%
	Con 5	102479	101816	99807	82913	34806645	419.8	52.73%	98.49%	94.85%
	Con 6	105585	104823	102423	80739	33900938	419.88	55.47%	98.32%	94.40%
NPs	NPs 1	74506	73930	71859	47075	19719915	418.9	53.66%	98.35%	94.39%
	NPs 2	115999	115314	112119	91235	38005976	416.57	54.86%	98.19%	94.17%
	NPs 3	104132	103363	100173	71403	29946031	419.39	53.68%	98.15%	93.88%
	NPs 4	93786	93073	90236	59386	24893669	419.18	53.59%	98.23%	94.19%
	NPs 5	105672	105021	101802	67789	28280415	417.18	54.11%	98.29%	94.33%

NPs 6	109898	109075	105967	65229	27183163	416.73	54.07%	98.22%	94.20%

Table S3. 16s rRNA averaged relative abundance of the top 20 main intestinal microbial colonies at the genus level.

Taxonomy	Con Group	NPs Group
Bacteroides	0.146764	0.198512
Rikenellaceae_RC9_gut_group	0.026802	0.068259
Prevotellaceae_UCG-001	0.083829	0.022177
Faecalibacterium	0.040072	0.053019
Megamonas	0.054315	0.044462
Phascolarctobacterium	0.050255	0.041216
Methanobrevibacter	0.050605	0.011079
Enorma	0.029902	0.002319
Fusobacterium	0.014446	0.027983
Lactobacillus	0.012629	0.022406
Desulfovibrio	0.02522	0.032455
[Ruminococcus]_torques_group	0.020316	0.020741
Olsenella	0.01834	0.004193
Synergistes	0.021205	0.00674
Prevotellaceae_NK3B31_group	0.007197	0.000451
unidentified_Saccharimonadales	0.011701	0.004993
Anaerosporobacter	0.001734	0.005006
Barnesiella	0.002052	0.008697
Alistipes	0.0085	0.006016
CHKCI001	0.003716	0.00998
Others	0.3704	0.409296

Note: Average relative abundance.

Table S4. Relative abundance of 16s rRNA clustering heatmap.

Taxonomy	Con1	Con2	Con3	Con4	NPs1	NPs2	NPs3	NPs4
Bacteroides	0.14300961	0.17263811	0.16351578	0.10796869	0.20513798	0.20729786	0.22475479	0.1574427
Rikenellaceae_RC9_gut_group	0.0298826	0.01923566	0.02624892	0.03178838	0.13053311	0.06421202	0.02757026	0.0499568

Prevotellaceae_UCG-001	0.0641612	0.09384052	0.06627026	0.11071302	0.02734157	0.02411445	0.00780099	0.02973014
Faecalibacterium	0.04855923	0.05686843	0.03140723	0.02360624	0.042105	0.036718	0.03244905	0.10085379
Megamonas	0.06723586	0.0379631	0.0866748	0.02513086	0.00919856	0.07356304	0.05940946	0.03554912
Phascolarctobacterium	0.04886416	0.0235046	0.07968694	0.04878792	0.04967729	0.04523047	0.0433501	0.02642679
Methanobrevibacter	0.06843015	0.0298826	0.03171215	0.0722417	0.00015246	0.02091274	0.00899527	0.01417899
Enorma	0.00459928	0.02642679	0.05905372	0.02975555	0.005387	0.00101642	0.00165167	0.00129593
Fusobacterium	0.00241399	0.01123139	0.01961681	0.02436855	0.01092646	0.02462266	0.02861209	0.0480002
Lactobacillus	0.0195914	0.01824465	0.00162626	0.01090105	0.01430604	0.04497637	0.02045535	0.00985923
Desulfovibrio	0.00581898	0.02833257	0.03153428	0.03493927	0.04474767	0.02952686	0.03148346	0.02380952
[Ruminococcus]_torques_group	0.01677085	0.03626061	0.02368247	0.00444682	0.01407735	0.01857499	0.03128017	0.01928648
Olsenella	0.00891904	0.00863953	0.03382121	0.02200539	0.0051329	0.00345581	0.00477715	0.00307466
Synergistes	0.00614931	0.02683336	0.02754485	0.02434314	0.01489048	0.00622554	0.00386238	0.00172791
Prevotellaceae_NK3B31_group	0.00094018	0.00055903	5.08E-05	0.02731616	2.54E-05	0.00012705	0	0.00165167
unidentified_Saccharimonadales	0.02413986	0.00973217	0.00996087	0.00307466	0.00282055	0.00045739	0.00030492	0.01644051
Anaerosporobacter	0.00688621	0	0	0	0.00083854	0.01804137	0.0007369	0.0002541
Barnesiella	0.00452305	0.00218529	0.00068608	0.00086395	0.00388779	0.01697413	0.00914774	0.00482797
Alistipes	0.00571733	0.00866494	0.00292219	0.01677085	0.00830919	0.00467551	0.00411648	0.00675916
CHKCI001	0.00767393	0.00261727	0.00160085	0.00284596	0.01400112	0.00355745	0.00576816	0.0164151

Note: Average relative abundance.

Table S5. 16s rRNA relative circos of the top 20 main intestinal microbial colonies at the genus level.

Taxonomy	Con	NPs	Tax_detail
Bacteroides	0.130172	0.182563	k__Bacteria; p__Bacteroidota;c__Bacteroidia;o__Bacteroidales;f__Bacteroidaceae;g__Bacteroides;
Megamonas	0.082762	0.04148	k__Bacteria; p__Firmicutes;c__Negativicutes;o__Veillonellales-Selenomonadales;f__Selenomonadaceae;g__Megamonas;
Rikenellaceae_RC9_gut_group	0.026432	0.062825	k__Bacteria; p__Bacteroidota;c__Bacteroidia;o__Bacteroidales;f__Rikenellaceae;g__Rikenellaceae_RC9_gut_group;
Prevotellaceae_UCG-001	0.088606	0.029044	k__Bacteria; p__Bacteroidota;c__Bacteroidia;o__Bacteroidales;f__Prevotellaceae;g__Prevotellaceae_UCG-001;
Faecalibacterium	0.033994	0.044158	k__Bacteria; p__Firmicutes;c__Clostridia;o__Oscillospirales;f__Ruminococcaceae;g__Faecalibacterium;
Phascolarctobacterium	0.047482	0.039493	k__Bacteria; p__Firmicutes;c__Negativicutes;o__Acidaminococcales;f__Acidaminococcaceae;g__Phascolarctobacterium;
Methanobrevibacter	0.048564	0.013168	k__Archaea; p__Euryarchaeota;c__Methanobacteria;o__Methanobacteriales;f__Methanobacteriaceae;g__Methanobrevibacter;
Enorma	0.027443	0.002856	k__Bacteria; p__Actinobacteriota;c__Coriobacteriia;o__Coriobacteriales;f__Coriobacteriaceae;g__Enorma;
[Ruminococcus]_torques_group	0.017401	0.026589	k__Bacteria;p__Firmicutes;c__Clostridia;o__Lachnospirales;f__Lachnospiraceae;g__[Ruminococcus]_torques_group;
Fusobacterium	0.012161	0.022371	k__Bacteria; p__Fusobacteriota;c__Fusobacteriia;o__Fusobacteriales;f__Fusobacteriaceae;g__Fusobacterium;
Lactobacillus	0.015531	0.020374	k__Bacteria; p__Firmicutes;c__Bacilli;o__Lactobacillales;f__Lactobacillaceae;g__Lactobacillus;
Desulfovibrio	0.023225	0.028409	k__Bacteria; p__Desulfobacterota;c__Desulfovibrionia;o__Desulfovibrionales;f__Desulfovibrionaceae;g__Desulfovibrio;
Olsenella	0.022087	0.00556	k__Bacteria; p__Actinobacteriota;c__Coriobacteriia;o__Coriobacteriales;f__Atopobiaceae;g__Olsenella;
Synergistes	0.021492	0.006002	k__Bacteria; p__Synergistota;c__Synergistia;o__Synergistales;f__Synergistaceae;g__Synergistes;
unidentified_Saccharimonadales	0.013274	0.008838	k__Bacteria; p__Patescibacteria;c__Saccharimonadia;o__Saccharimonadales;f__unidentified_Saccharimonadales;g__unidentified_Saccharimonadales;
Alistipes	0.011343	0.00742	k__Bacteria; p__Bacteroidota;c__Bacteroidia;o__Bacteroidales;f__Rikenellaceae;g__Alistipes;

Collinsella	0.010337	0.001987	k__Bacteria; p__Actinobacteriota;c__Coriobacteriia;o__Coriobacteriales;f__Coriobacteriaceae;g__Collinsella;
Others	0.36769	0.456852	Others
